# Resistance to insect pests in wheat—rye and *Aegilops speltoides* Tausch translocation and substitution lines

**DOI:** 10.1007/s10681-019-2449-7

**Published:** 2019-06-18

**Authors:** L. A. Crespo-Herrera, R. P. Singh, A. Sabraoui, M. El-Bouhssini

**Affiliations:** 1 Global Wheat Program, Centro Internacional de Mejoramiento de Máız y Trigo (CIMMYT), Apdo. 0660, Mexico, DF, Mexico; 2 The International Center for Agricultural Research in the Dry Areas (ICARDA), Rabat, Morocco

**Keywords:** Russian wheat aphid, Hessian fly, Stem sawfly, Resistance, Wheat

## Abstract

Various insect pests attack wheat (*Triticum aestivum* L.) that can cause significant grain yield losses to the crop. Farmers usually depend on pesticides, however, smallholder farmers often have limited and ill-timed access to control methods, including insecticides. Host plant resistance is an alternative to protect grain yield and reduce costs to farmers. Three of the most serious pests of wheat are *Diuraphis noxia* (Kurdjumov), *Mayetiola destructor* (Say), and *Cephus pygmeaus* L. These pests occur in most of the wheat growing areas. However, they are of high importance in North Africa and West Asia. The aim of this study was to evaluate a set of wheat—alien translocations for resistance against *D. noxia, M. destructor* and *C. pygmeaus*. Genotypes of this germplasm set have already been reported to carry resistance against certain wheat aphid species. Genotypes 1RS_am_.1AL and MA1S.1RL_e_(1B), displayed high levels of resistance against *D. noxia* and *C. pygmeaus*, respectively. While three genotypes showed resistance reaction against *M. destructor*: 1R_e_(1D), 7A.7S-L5, and 7A.7S-Gb5. Except for the resistant genotype to *C. pygmeaus*, the other four genotypes were previously reported to carry resistance against *Sitobion avenae* Fabricius, *Rhopalosiphum padi* L. and *Schizaphis graminum* (Rondani). These five wheat—alien translocations are currently being used in the bread-wheat breeding programs at CIMMYT and ICARDA to transfer the multiple pest resistance in elite germplasm.

## Introduction

Wheat (*Triticum aestivum* L) is a staple food globally, which provides about 20% of energy intake in the human diet (FAO 2018). About one third of the harvested area globally (ca. 72 million hectares) is accounted by countries with developing economies in Central and South Asia, and East and North Africa, with an average yield of 2.3 t/ha (FAO 2018). Wheat production faces several challenges in a global scenario where climate change threatens productivity and higher food demand requires to increase average yields.

One of the effects of climate change is a higher incidence of pests, as with an increased temperature multivoltine species can speed up their development causing an increased number of generations per year, and potentially more damage to crops. There are various insect pests that can feed on wheat, among those that are highly important for the International Maize and Wheat Improvement Center (CIMMYT) and the International Center for Agricultural Research in the Dry Areas (ICARDA) target environments are, the Russian wheat aphid (*Diuraphis noxia* Kurdjumov), the Hessian fly (*Mayetiola destructor* [Say]) and the wheat stem sawfly (*Cephus* spp.).

The aphid species *D. noxia*, is believed to originate from Central Asia, between Caucasus Mountains and the Tian Shan (Berzonsky et al. 2003). It can reduce yield up to 40% at an initial density of 15 aphids (Kieckhefer and Gellner 1992). This aphid species injects a toxin into plants while feeding, causing a characteristic leaf rolling, which functions as a protection site for the colony. When the flag leaves are infested and rolled, the heads are trapped and cannot emerge freely thus causing them to bend, also, the leaves get white, purple and yellow streaks. *Diuraphis noxia* is widely distributed as a pest in East Asia, South Africa and North and South America, central Europe, North Africa, the Middle East and Australasia (Berzonsky et al. 2003; Blackman and Eastop 2007; Zhang et al. 2014; Yazdani et al. 2017).

The Hessian fly, *M. destructor*, is an introduced pest in the American continent first observed in the late 1770 s, however, its origin is thought to be the West Asia (Naber et al. 2000). This insect is a serious pest in North Africa: Morocco, Algeria and Tunisia (Berzonsky et al. 2003). However, it is also present in Central Asia, South Europe and North America. A population of *M. destructor* that originated from Syria is reported to be virulent to most of the resistance genes identified, being avirulent only on genes *H25* and *H26* (El Bouhssini et al. 2009). The larvae of the fly feeds on the stems of young plants which prevents elongation of internodes and transport of nutrients causing significant yield losses up to 40% (Smiley et al. 2004; Beres et al. 2011).

There are several species of the *Cephus* genus that attack wheat, the most common in North America is *Cephus cinctus* Norton (Beres et al. 2011). However, the predominant species in North Africa and West Asia is *Cephus pygmaeus* L. (Golberg 1986; El Bouhssini et al. 1987). This species, *C. pygmaeus*, is also reported to be present in Europe and Central Asia (Leach and Hobbs 2013; CABI-EPPO 2018). The adults of *Cephus* spp. are univoltine, they oviposit into elongating stems of the plant, when the eggs hatch, thelarvae feed within the stem moving in, up and down. As the plant reaches maturity, the larvae move to the basal part of the plant to build an hibernaculum, above of which the plant weakens and breaks. Then the larvae go into diapause during the winter (Golberg 1986; Shanower 2008).

Host plant resistance (HPR) is an environmentally friendly method to control insect pests. When HPR is present in commercial varieties, farmers can benefit because they can reduce the insecticide usage, and subsequently the production costs and negative effects on the environment and non-targeted organisms. Farmers that have limited access to other control methods can make use of HPR by simply sowing the seeds of varieties that carry the genes for resistance against important pests, and subsequently, protect yield in the occurrence of pest outbreaks. However, screening for insect HPR is time consuming and labor intensive, which makes it difficult to implement phenotypic selection methods in large breeding programs to develop elite germplasm with insect resistance.

Our study aimed to identify resistance sources to *D. noxia, M. destructor* and *C pygmaeus* in a set of wheat—alien translocations and substitution lines that had been previously evaluated for resistance against three aphid species. From our evaluations, we indicate which plant genotypes carry resistance to each of these pests and the implications for further wheat breeding and research.

## Materials and methods

### Plant material

The plant materials consisted of a set of 62 wheat—rye and wheat—*Aegilops speltoides* Tausch translocations produced after 6–8 backcrosses to the spring bread-wheat cultivar Pavon F76 (Lukaszewski 1993, 1997, 2000, 2006; Dubcovsky et al. 1998). This material was selected because it displayed variation of the resistance against other important pests of wheat, further description of the germplasm can be found in the work conducted by Crespo-Herrera et al. (2013).

### Screenings

Evaluations to *M. destructor* and *D. noxia* were conducted under greenhouse conditions at 20–22 °C, photoperiod of 16:8 h (light:dark), and a relative humidity of 60–70%. Because the objective of the study was to identify resistant germplasm, and due to the fact that for insect resistance under high and homogeneous insect pressure the rate of false negatives is practically null, we conducted unreplicated tests. Only those lines that displayed resistant reactions were further evaluated in replicated tests to confirm the resistance.

#### M. destructor

The Hessian fly individuals originated from a population collected in the Chaouia region, Morocco. It was reared and increased on the susceptible cv ‘Radia’ under the same conditions described above (El Bouhssini et al. 2013). The screening was conducted in a greenhouse. The initial screening was carried out in a standard greenhouse flat (54 cm 9 36 cm 9 8 cm) containing a mixture of soil, vermiculite and peat. At the one leaf stage, each screening flat was covered with a cheesecloth tent where about 50 mated females were released and allowed to lay eggs for 2 days. Twenty seeds of each plant genotype were sown in rows, and the percentage of resistant plants was taken from each row. Resistant plants were those that remained healthy while the susceptible check was dead due to insect damage. The resistant check for this evaluation was cv. ‘Arrehane’ and the susceptible check was cv ‘Radia’. Lines displaying resistance were reevaluated following the same procedure but with four replications.

#### D. noxia

Individuals of D. noxia were collected from the Annoceur region, in the middle Atlas of Morocco. Aphid rearing was done in the greenhouse under the same conditions as the evaluations. The aphids were reared for 3–4 generations to make sure there are no parasitoids in the RWA culture (El-Bouhssini et al. 2011). Seeds were planted in tufts in flats with five seeds per tuft, which were thinned to three plants per tuft after germination. Seeds were sown in a mixture of soil, sand and peat (2:1:6). Each plant was infested with 10 adult RWA at the two-leaf stage. Evaluations were made when the susceptible check displayed maximum level of damage on the scales of 1–3 for leaf rolling (LR) and 1–6 for leaf chlorosis (LC), where the lowest number indicates fully resistant plants, i.e., both leaf rolling and chlorosis absent. Plant genotypes that displayed resistance reactions were further tested, following the same procedures, in a four-rep evaluation. The susceptible check for this test was cv. ‘Achtar’.

#### C. pygmaeus

The screening for resistance to wheat stem sawfly was done under field conditions under natural infestations during two crop cycles, first at Merchouch station (2015/16) and then at Sidi El Aidi station (2016/17) in Morocco. Genotypes were planted in 1.0 m plots in three replicated randomized blocks during each crop cycle. Average percentage of stem cut in the three reps was taken as a measure of resistance; lines with less than 5% stem cut are considered as resistant, given that 10% is considered the economic threshold (Özberk et al. 2005). The material was scored just before harvest. The resistant check for this evaluation was line ‘AWYT-01-LR-405’, and the susceptible check was cv. Achtar.

## Results and discussion

The vast majority of the germplasm we evaluated showed low or null levels of resistance against *D. noxia, M. destructor* and *C. pygmaeus* (Table 1). There were, however, five lines that displayed exceptional levels of resistance against these pests, but none against all three pests together. High levels of resistance were displayed in two single genotypes, 1RS_am_.1AL and 1MA1S.1RL_e_(1B), against *D. noxia* and *C. pygmaeus* respectively (Table 1). While three genotypes showed resistance reaction against *M. destructor*: 1R_e_(1D), 7A.7S-L5, and 7A.7S-Gb5.

**Table 1:** Damage of insect pests on wheat—rye and wheat—*A. speltoides* translocation lines

Genotype	WSSF	HF	RWA	
	% Stems cut	% Resistant plants	Leaf rolling	Leaf chlorosis
1R_e_(1B)	16	0	3	4
1R_e_(1D)	20	100^a^	3	4
MA1S.1RL_e_(1A)	20	0	3	4
MA1S.1RL_e_(1B)	0	0	3	4
MA1S.1RL_e_(1D)	12	0	3	4
1R_pr_(1D)	28	0	3	6
1R_pr_.1D5 + 10 - 2(1D)	24	0	3	4
1R_rec_(1A)	40	0	3	6
1R_rec_(1B)	40	0	3	6
1R_inv_(1A)	20	0	2	4
2R_rec_(2B)	20	0	3	4
1RS_e_.1AL	16	0	3	4
1RS_am_.1AL	20	0	1^a^	1^a^
1RS_v_.1AL	16	0	2	4
1RS_rh_.1AL	12	0	3	4
1RS_e_.1BL_v_	16	0	2	4
1RS_cim_.1BL	24	0	3	4
1RS.1BL_gnr_	16	0	3	4
1RS_v_.1BL	16	0	3	4
1RS_i_.1BL	16	0	3	4
1RS_i_.1BL	16	0	3	4
MA1	12	0	2	4
MA2	20	0	3	4
Te1	12	0	2	4
Te2	12	0	2	4
1B_ins_	20	0	3	4
1R_i_(1B)	28	0	3	6
1RS_bb_.1DL	12	0	3	4
1RS_w_.1DL	12	0	3	4
1RS_e_.1DL	20	0	3	4
1RS_v_.1DL	24	0	3	4
1AS.1RL_e_	16	0	2	4
1BS.1RL_e_	16	0	2	4
1DS.1RL_e_	12	0	2	4
1DS.1RL_bb_	16	0	2	4
1AS.#2L	16	0	3	4
1RS.1AL″ 1RS.1DL″	16	0	3	4
2RS_cs_.2BL	12	0	2	4
2AS.2RL_cs_	20	0	3	6
2BS.2RL_cs_	16	0	3	4
2BSp.2RL_bl_	12	0	3	4
2BS.2RL_cs_	12	0	3	6
3RS_rh_.3DL	16	0	3	4
3RS.3DL_cs_	12	0	3	4
4A_ril_	20	0	3	4
5RS.5AL_cs_	32	0	3	4
5RS_e_.5BL	16	0	3	4
5RS_rh_.5DL	20	0	3	4
6BS.6RL_bb_	20	0	3	4
7DS.4RL_m_	20	0	3	4
1D + 9″	12	0	3	4
T-9″	24	0	3	4
1D + 4″	16	0	3	4
2D(s) + 2″	16	0	3	4
2D(s) + 4″	20	0	3	4
2R.2B	16	0	3	6
5D.5R-1″	24	0	3	4
7A.7S-S3	16	0	3	6
7A.7S-L7	12	0	3	4
7A.7S-L5	20	100^a^	3	4
7A.7S-Gb5	16	100^a^	3	6
Resistant check	4	100	-	-
Susceptible check	20	0	3	6

a Scores derived from replicated test for the case of D. noxia and M. destructor

Interestingly, the genotype 1RS_am_.1AL resistant against *D. noxia* carries the chromosome arm from ‘Insave’ rye, the same that is carried by cv. ‘Amigo’. In previous studies, this line was resistant to S. avenae in seedling and adult plant tests (Crespo-Herrera et al. 2013). Since the *Dn7* gene for *D. noxia* resistance is on chromosome arm 1RS (Anderson et al. 2003), it would be relevant to determine if this is related to the resistance in 1RS_am_.1AL. Previous investigations have reported Amigo wheat derivatives as susceptible to *D. noxia*, i.e., lines carrying the chromosome arm 1RS (Webster et al. 1987). The gene *Dn7* confers high levels of resistance against D. noxia populations collected in Syria, and biotypes RWASA1, RWASA2 and RWASA3 in South Africa (El-Bouhssini et al. 2011; Jankielsohn 2011). This gene is also the only one effective against emerged *D. noxia* biotypes from the USA (Haley et al. 2004). The 1R translocation present in cv. Amigo also carries *Gb2* for *S. graminum* biotype B resistance, although the most common biotypes are E and I. Furthermore, it also carries the Cmc3 gene for resistance to Aceria tosichella Keifer and Pm8 for resistance against *Blumeria graminis* (DC.) Speer *f. sp. tritici* (Crespo-Herrera et al. 2017). This particular 1RS translocation is also reported to have certain yield advantage compared with Pavon F76, i.e., Pavon F76 without the translocation (Kim et al. 2004). An additional consideration of relevance for the use of the 1RS_am_.1AL translocation is that it causes the least detrimental effects on industrial quality, which is an advantage over 1B/1R translocations (Kumlay et al. 2003), carried by those genotypes with the *Dn7* gene.

The genotypes resistant to *M. destructor* are also reported to possess resistance to other pests. For instance, 1R_e_(1D) carries resistance to *R. padi* and *S. avenae* at seedling stages (Crespo-Herrera et al. 2013). Although, resistance to *S. avenae* at seedling stages may not be relevant since this aphid species attacks wheat during reproductive stages (Watt 1979; Voss et al. 1997). The genotypes 7A.7S-L5, and 7A.7S-Gb5 are wheat-*A. speltoides* interstitial translocations, and reportedly to carry resistance against *S. graminum* (Dubcovsky et al. 1998; Crespo-Herrera et al. 2013). Interestingly, in the report of Crespo-Herrera et al. (2013) another related genotype (7A.7S-L7) derived from the same original translocation carrying the gene *Gb5*, did not display resistance against *M. destructor*. These A. speltioides translocations are located towards the 15% distal section of chromosome arm 7AL, however they have different breakpoints (Lukaszewski 1995), which is the most likely reason why 7A.7S-L5 is resistant to *M. destructor* but 7A.7S-L7 is not. El-Bouhssini et al. (2008) evaluated 278 accessions of different Aegilops species, of which 18 were *A. speltoides* accessions, and two of these were reported to be resistant to *M. destructor*. Work is ongoing to develop easy to use molecular markers that can aid the selection for these *A. speltoides* translocations.

The genotype MA1S.1RL_e_(1B), resistant to *C. pygmaeus*, carries an engineered 1R chromosome from two sources (Lukaszewski 2006). This line did not show any significant level of resistance in previous studies (Crespo-Herrera et al. 2013). Resistance to this wasp is correlated with stem solidness and plant earliness (Varella et al. 2015), and genomic regions on chromosomes 1B, 3B and 5D for stem solidness, and 2A, 3A, 5B for antibiosis to the larvae have been reported (Varella et al. 2015). In a separate study Joukhadar et al. (2013) found associations to stem cut on chromosomes 1D, 3B, 5B, 6B and 7A.

Translocation and substitution lines carrying the 1R chromosome from rye are well known to cause deleterious effects on quality, in particular when 1R substitutes chromosome 1B of wheat. When the translocations is 1RS.1AL, the effects on quality are not as detrimental as 1B(1R) substitution lines (Kumlay et al. 2003). Nonetheless, the effect on agronomic performance of the 1R chromosome appears to be dependent on the source and the background into which it is transferred (Kim et al. 2004).

Wheat-alien translocations can harbor stress resistance/tolerance genes, and their identification is possible through cytogenetic procedures or molecular markers. The advantage of these translocations is that they normally do not recombine with wheat chromosomes in the presence of the *Ph1* gene. Hence when they carry genes for resistance to more than one pest or disease, this resistance can be inherited simultaneously, thus the importance of screening this type of materials against various pests or diseases. Translocations, however, may have the disadvantage of linkage drag, especially when large chromosomal segments are transferred from the alien species, and their expression can also be determined by other loci in the genome that may act as suppressors. Breeding efforts are underway to transfer multiple pest resistance to elite germplasm at CIMMYT and ICARDA wheat breeding programs by utilizing the sources reported in this work and other previously reported in literature.

## Notes

**Publisher’s Note** Springer Nature remains neutral with regard to jurisdictional claims in published maps and institutional affiliations.
